# On Reuben G. Jones synthesis of 2-hydroxypyrazines

**DOI:** 10.3762/bjoc.18.93

**Published:** 2022-07-29

**Authors:** Pierre Legrand, Yves L Janin

**Affiliations:** 1 Synchrotron SOLEIL, L'Orme des Merisiers, 91190 Saint-Aubin, France; 2 Structure et Instabilité des Génomes (StrInG), Muséum National d'Histoire Naturelle, INSERM, CNRS, Alliance Sorbonne Université, 75005 Paris, France

**Keywords:** α-aminoamide, condensation, hydroxypyrazine, methylglyoxal, phenylglyoxal

## Abstract

In 1949, Reuben G. Jones disclosed an original synthesis of 2-hydroxypyrazines involving a double condensation between 1,2-dicarbonyls and α-aminoamides upon treatment with sodium hydroxide at low temperature. This discovery turned out to be of importance as even today there are no simple alternatives to this preparation. Across the years, it was employed to prepare 2-hydroxypyrazines but some of its limits, notably regioselectivity issues when starting from α-ketoaldehydes, certainly hampered a full-fledged generation of pyrazine-containing new chemical entities of potential interest in medicinal chemistry. The present text describes some insights and improvements, such as the unprecedented use of tetraalkylammonium hydroxide, in the reaction parameters affecting the regioselectivity and yield when starting from phenylglyoxal and two α-aminoamides. We also suggest a mechanism explaining the counterintuitive occurrence of 3,5-substituted-2-hydroxypyrazine as the major reaction product.

## Introduction

**Scheme 1:**
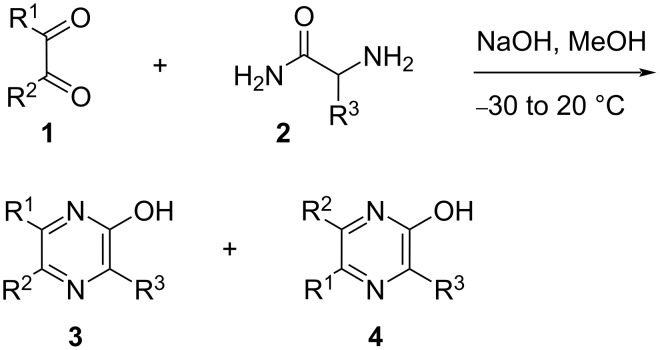
Reuben G. Jones synthesis of 2-hydroxypyrazines.

In a recent report [1], the many ways a pyrazine nucleus is able to interact with proteins were reviewed. In this text, it was also mentioned that this heterocycle appears to be under-represented in the DrugBank database (in comparison with pyridine-containing substances) plausibly because of a lesser “availability of pyrazine fragments in vendors”. For chemists, this statement could be translated as “fragments” far more difficult to make due to the lack of simple and general synthetic pathways to compounds featuring this heterocycle. This aspect, which could be considered as another “chemical blind spot” [2], was actually amongst the motivations of notable chemistry-oriented approaches which used palladium-catalyzed reactions to generate libraries of pyrazine derivatives [3–4]. In any case, as depicted in Scheme 1, Reuben G. Jones, working in 1949 in Eli Lilly’s Indianapolis research facilities, reported a sodium hydroxide-promoted condensation between 1,2-dicarbonyls **1** and the free base of α-aminoamides **2** to give the 2-hydroxypyrazines **3** and/or **4** [5–6]. This discovery, possibly inspired by the contemporary base-promoted condensation between phenylglyoxal and aminoguanidine to give 3-amino-1,2,4-triazines [7–8]. remains on paper, the easiest access to 2-hydroxypyrazines [9–12]. Moreover, this synthesis was simplified later on by the use of the hydrochloride salts of the α-aminoamides **2**, instead of their free base, along with the addition of a second equivalent of sodium hydroxide [13]. As expected, from asymmetric 1,2-diketones (**1**, R^1^ ≠ R^2^ and ≠ H), reports [14–16] describe the occurrence of both isomers **3** and **4** and a more recent publication is mentioning that this is a recurrent issue [17]. On the other hand, a fascinating aspect of this reaction is seen when starting from α-ketoaldehydes (**1**, R^1^ = H, R^2^ ≠ H). Indeed, if only an average reaction yield is usually obtained, it is the least expected 3,5-substituted-2-hydroxypyrazine isomer (**3**, R^1^ = H, R^2^ ≠ H) which is isolated sometimes along with a much smaller amount of the alternative 3,6-substituted-2-hydroxypyrazines (**4**, R^1^ = H, R^2^ ≠ H) [6,13,18–28].

**Scheme 2:**
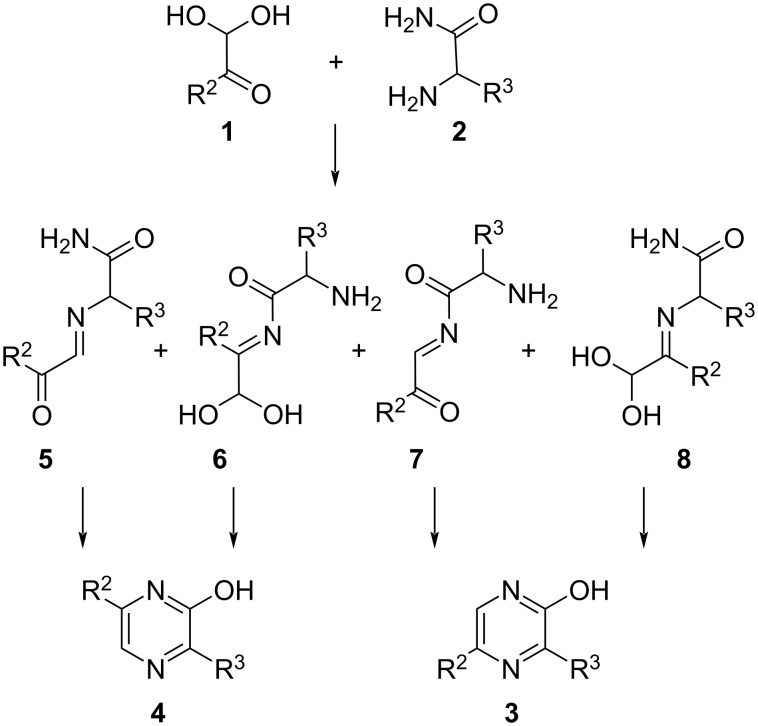
Four hypothetical reaction intermediates.

This regioselectivity appears counterintuitive when considering Scheme 2. The first condensation between α-ketoaldehyde **1** (depicted in its hydrated form) and α-aminoamide **2** could lead to the four different products **5**–**8**. The most likely to occur would be the one resulting from the condensation of the most nucleophilic group of the α-aminoamide (its amine) on the most electrophilic component of the α-ketoaldehyde (its aldehyde) to give intermediate **5**. However, the ensuing cyclization (via a hydration of its imine bond to allow for a rotation) would then lead to compound **4** which is rarely the major reaction product. Since compound **3** is the main result of this reaction, then the less likely occurrence of intermediates **7** and/or **8** could account for this result. However, since from α-ketoaldehydes, this reaction is achieved in the presence of sodium hydroxide, a far more complex reaction mechanism is likely; although to the best of our knowledge, this has never been the subject of a report. Our recent interest in the preparation of 3,5-substituted-2-hydroxypyrazines (**3**, R^2^ = Ar and R^3^ = CH_2_Ar) as intermediates for the synthesis of marine luciferins analogues [29–30] along with the simplicity of this access drove us to study some of its aspects which led to the improvements and insights described in the following.

## Results and Discussion

The preparation of 3,5-substituted-2-hydroxypyrazines **3** is usually undertaken [6,13,18–23,25–28,31] as follows. The α-ketoaldehyde **1** and the hydrochloride salt of the α-aminoamide **2** are dispersed in methanol, the suspension is cooled (usually at −30 °C) and a concentrated solution of sodium hydroxide is added. The resulting solution is allowed to warm back to room temperature and stirred, usually for few hours, before undertaking a workup procedure often initiated with the addition of concentrated hydrochloric acid. When relevant, this is followed by a dilution in an excess of water and then a filtration and washing. This procedure provides average yields of the 3,5-substituted-2-hydroxypyrazine **3** isomer as the major reaction product sometimes along with lesser amounts of the corresponding 3,6-substituted isomer **4**. Of note is a 1978 report, focusing on the synthesis of hydroxypyrazine itself, which has demonstrated that the rate of addition of sodium hydroxide is of importance as well as the temperature of the solution in the course of this addition. These observations were made on a 0.2 mol scale and an optimal addition rate of a 12 N sodium hydroxide solution of 8.0–8.6 mmol/min was reported [32]. For our part, instead of starting the reaction at −30 °C, we used the lower temperature of −78 °C which only requires a cooling bath containing dry ice and ethanol. Moreover, since no detectable transformations took place when quenching the reaction at −78 °C (at least after three hours when using phenylglyoxal and phenylalanine amide), the reactions were always allowed to warm to room temperature by removal of the dry ice bath. In all our trials, the two isomers **3** and **4** always occurred although in a ratio dependent on the reaction conditions used. Since the (minor) isomer **4** sometimes turned out to be, to some degree, soluble in acid aqueous phase and could thus be washed away by filtration, we undertook whenever possible a solvent extraction of the reaction products. One aspect of 2-hydroxypyrazine chemistry, which may further complicate a full-fledged study of this reaction, is that some derivatives (i.e., 5-methyl-2-hydroxypyrazine) appears to be not stable, especially in the presence of acid [33]. Concerning the purification of our reaction trials, as previously reported [13], the separation (by any mean) of the 2-hydroxypyrazine isomers **3** and **4** is challenging. To overcome this, we used water-heated columns and preheated eluent mixtures (see the experimental part) which allowed to run chromatography over silica gel at 60 °C. At this temperature and using relevant mixtures of cyclohexane and ethyl acetate, the complete elution of the less polar but also much less soluble 3,6-substituted isomers **4**{1,1} or **4**{1,2} took place before the occurrence of the fraction containing the more polar 3,5-substituted 2-hydroxypyrazines **3**{1,1} or **3**{1,2}. As listed in Table 1 and Table 2, we undertook optimization trials focusing on the condensation between phenylglyoxal (**1**{1}) and the hydrochloride salts of either alanine amide (**2**{1}) or phenylalanine amide (**2**{2}). In the course of this, many reaction parameters were taken into account (i.e., base addition speed (s), temperature (*T*), dilution (N), number of equivalents (equiv), and nature of the base (B) used in the course of the first step, solvent (S) used, reaction duration (*t*), and quench procedure) and the following is only a condensed account of this. Concerning the speed of the base addition, as previously reported [32], this parameter turned out to be essential. As seen in entries 1 and 2 of Table 1, even at −78 °C, a fast addition (5.5 mL of a 6 N solution in less than 20 seconds) in comparison with a slow one (same solution but in 8–9 minutes), had a real incidence on the yields of isolated compounds **3** and **4**. As suggested before [32], we also believe that a fast addition is causing local temperature increase in the reaction medium which is detrimental to the reaction yield at this stage (see the disastrous results of a trial at 20 °C described in entry 16 in Table 2). To avoid the use of an automated syringe injector, these slow additions were undertaken by using a syringe fitted with its 0.8 mm needle but devoid of its piston so that the rate of the addition was governed solely by gravity and the viscosity of the solution added. Thus, the addition of 5.5 mL of a 6 N solution of sodium hydroxide took place in 9 minutes and thus corresponded to an addition rate of 3.6 mmol/min. Dilution of the base (from 6 N to 2 N; Table 1, entries 2 and 3) had little or no effect on the reaction yield although the addition rate decreased a bit. Shortening the ensuing reaction time from two hours to one (Table 1, entries 2 and 4) led to a slight drop of the reaction yield but a longer time (4 hours) had no impact (Table 1, entry 5). A neutralization of the reaction medium with ammonium chloride (instead of 37% hydrochloric acid), followed by the addition of an excess of water, also caused a drop of the yield, plausibly because of a degree of water solubility of the ammonium salts of compound **3**{1,1} and **4**{1,1}. The effect of using triethylamine instead of sodium hydroxide as a base was remarkable. At room temperature, after an 18 hours-long reaction, hydroxypyrazine **4**{1,1} was the sole isomer detected and, out of complex reaction mixture, was isolated in a 11% yield (Table 1, entry 7). At reflux, the same isomer was also obtained although in an 8% yield (Table 1, entry 8). Also remarkable is the use of 1.5 equiv of sodium hydroxide instead of 2.5 (Table 1, entry 9). This also led to substantial changes in the yields of isomers **3**{1,1} and **4**{1,1} which illustrates the crucial effect of adding an excess of a strong base on the regioselectivity of this double condensation; before allowing the reaction to warm back to room temperature. These notable shifts of reaction regioselectivity should be taken into account when considering previous reports [6,13,31], which used either triethylamine as a base or lesser amounts of sodium hydroxide and sometimes did/could not provide a full structural attribution of the resulting reaction product. Out of results further described in Table 2, we also used a 20% tetrabutylammonium hydroxide solution instead of sodium hydroxide as a base (Table 1; entry 10) but, in this case and as compared with entry 3, only a marginally greater 76% yield of compound **3**{1,1} was obtained. Another puzzling result found in the course of these trials, is the yield of isolated compound **3**{1,1} observed when changing from a 1 to 1 proportion of the reactants **1**{1} and **2**{1} to a 2 to 1 (Table 1, entry 11) or 1 to 2 (Table 1, entry 12). In both cases, the yields were amongst the best obtained but in the presence of an excess of phenylglyoxal (**1**{1}), an intriguing 85% yield (in respect with the alanine amide (**2**{1})) was secured.

**Table 1:** Condensation trials between phenylglyoxal (**1**{1}) and alanine amide (**2**{1}).^a^

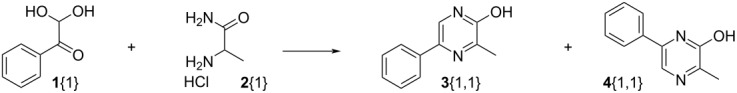										

entry	*T* (°C)	equiv	N	B	S	s	*t* (h)	comments	**3** [%]	**4** [%]

1	−78	2.5	6	NaOH	MeOH	65	2		52	4
2	−78	2.5	6	NaOH	MeOH	3.6	2		68	3
3	−78	2.5	2	NaOH	MeOH	2.3	2		71	4
4	−78	2.5	6	NaOH	MeOH	4.0	1		61	2
5	−78	2.5	6	NaOH	MeOH	4.6	4		70	4
6	−78	2.5	6	NaOH	MeOH	3.6	2	NH_4_Cl quench	60	1
7	20	2.5	–	NEt_3_	MeOH	–	18		0	11
8	reflux	2.5	–	NEt_3_	MeOH	–	22		0	8
9	−78	1.5	2	NaOH	MeOH	3.6	2	1.5 equiv of NaOH	34	18
10	−78	2.5	1.4	NEt_4_OH	MeOH	2.2	2		76	3
11	−78	2.5	2	NaOH	MeOH	3.6	2	1 equiv of **1**{1}, 0.5 equiv of **2**{1}	85	1
12	−78	2.5	2	NaOH	MeOH	3.6	2	0.5 equiv of **1**{1}, 1 equiv of **2**{1}	74	3

^a^Conditions: 1) stirring in solvent (S); 2) addition of base (B) with a (N) concentration, at speed s (mmol/min) and temperature *T* (°C); 3) stirring for *t* h (from *T* back to 20 °C); 4) addition of 4 equiv of 37% HCl; 5) workup (neutralization and extraction) and chromatography. All reactions run at a 13.1 mmol scale in 35 mL of solvent.

As listed in Table 2, some of the results of the reaction optimization studies using compounds **1**{1} and **2**{2} followed the trends reported in Table 1. But for entries 16 and 17 in Table 2, a slow addition of the base, at −78 °C, was always used and we also checked that an even slower addition (Table 2, entries 2 and 3) had no impact on the reaction yield. As illustrated in entry 16 (Table 2), addition of the sodium hydroxide at 20 °C led indeed to very poor results. Interestingly, the dilution of sodium hydroxide (added at −78 °C) had some impact on the reaction yield (Table 2, entries 5 and 6 in comparison with entry 2). Trials using ethanol or isopropanol as a solvent (Table 2, entries 7 and 8) were not successful, plausibly because of a precipitation of the reaction medium before the end of the base addition. Trials with lithium or potassium hydroxide (Table 2, entries 9 and 10) demonstrated a limited effect of this change (as compared with sodium hydroxide used in entry 5 of Table 2) and trials using ammonia or triethylamine (Table 2, entries 11 and 12) pointed out again the lack of occurrence of any isomer **3**{1,2} and, out of complex reaction mixtures, the occurrence of modest amounts of isomer **4**{1,2}. Of note is that the use of sodium ethoxide was not successful (Table 2, entry 13) whereas the use of 0.75 N tetrabutylammonium hydroxide either diluted in water or in methanol (Table 2, entries 14 and 15) had the biggest impact on the reaction yield. Interestingly, if yield and regioselectivity-wise a sodium hydroxide addition at 20 °C was disastrous (Table 2, entry 16), only a 10% yield loss (and no regioselectivity changes) was seen when using tetrabutylammonium hydroxide instead (Table 2, entry 17). Further trials using tetraethylammonium hydroxide (Table 2, entries 18–20) confirmed this previously unreported counter ion effect and pointed out that the dilution or the reaction duration had marginal effects. Moreover, changing the ratio of the reactants (Table 2, entries 21 and 22) had, as for entries 10 and 11 in Table 1, an impact on the reaction yield. Again, an excess of the α-ketoaldehyde **1**{1} led to the best reaction yield in regard with the α-aminoamide **2**{2}. Finally, since we often observed a degree of reaction medium freezing by the end of the base addition, a trial was made at −20 °C (Table 2, entry 23) and this gave the same yield as the one obtained at −78 °C (Table 2, entry 19).

**Table 2:** Condensation trials between **1**{1} and phenylalanine amide (**2**{2}).^a^

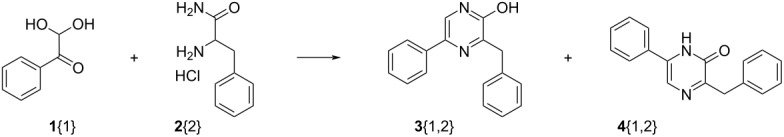										

entry	*T* (°C)	equiv	N	B	S	s	*t* (h)	comments	**3** [%]	**4** [%]

1	−78	2.05	6	NaOH	MeOH	2.9	2		40	4
2	−78	2.5	6	NaOH	MeOH	3.5	2		45	3
3	−78	2.5	6	NaOH	MeOH	0.5	2	very slow addition	45	3
4	−78	2.5	6	NaOH	MeOH	2.7	2	addition of H_2_O before HCl	45	5
5	−78	2.5	2	NaOH	MeOH	3.1	2		51	7
6	−78	2.5	1	NaOH	MeOH	1.7	2	freezing at −78 °C	58	3
7	−78	2.5	2	NaOH	EtOH	2.7	2	freezing at −78 °C	33	2
8	−78	2.5	2	NaOH	iPrOH	2.7	2	freezing at −78 °C	21	2
9	−78	2.5	2	LiOH	MeOH	1.6	2		56	6
10	−78	2.5	2	KOH	MeOH	3.1	2		54	4
11	−78	2.5	2	NH_4_OH	MeOH	3.0	2		0	6
12	reflux	2.5		NEt_3_	MeOH	–	18		0	25
13	−78	2.5	2.7	EtONa/EtOH	MeOH	2.0	2	1 mm needle	38	2
14	−78	2.5	0.75	NBu_4_OH	MeOH	0.9	2	base diluted in water, freezing at −78 °C	67	2
15	−78	2.5	0.75	NBu_4_OH	MeOH	0.7	2	base diluted in methanol	64	2
16	20	2.5	6	NaOH	MeOH	2.7	2	addition at 20 °C	11	8
17	20	2.5	1.4	NBu_4_OH	MeOH	75	2	fast addition at 20 °C	55	2
18	−78	2.5	2.8	NEt_4_OH	MeOH	1.1	2		61	2
19	−78	2.5	1.4	NEt_4_OH	MeOH	2.0	2		65	3
20	−78	2.5	1.4	NEt_4_OH	MeOH	2.0	3		66	3
21	−78	2.5	1.4	NEt_4_OH	MeOH	2.0	2	1 equiv of **1**{1}, 0.5 equiv of **2**{2}	77	2
22	−78	2.5	1.4	NEt_4_OH	MeOH	2.0	2	0.5 equiv of **1**{1}, 1 equiv of **2**{2}	70	4
23	−20	2.5	1.4	NEt_4_OH	MeOH	2.0	2	Addition at −20 °C	65	3

^a^Conditions: 1) stirring in solvent (S); 2) addition of base (B) with a (N) concentration, at speed s (mmol/min) and temperature *T* (°C); 3) stirring for *t* h (from *T* back to 20 °C); 4) addition of 4 equiv of 37% HCl; 5) workup (filtration) and chromatography. All reactions run at a 9.8 mmol scale in 26 mL of solvent.

**Figure 1:**
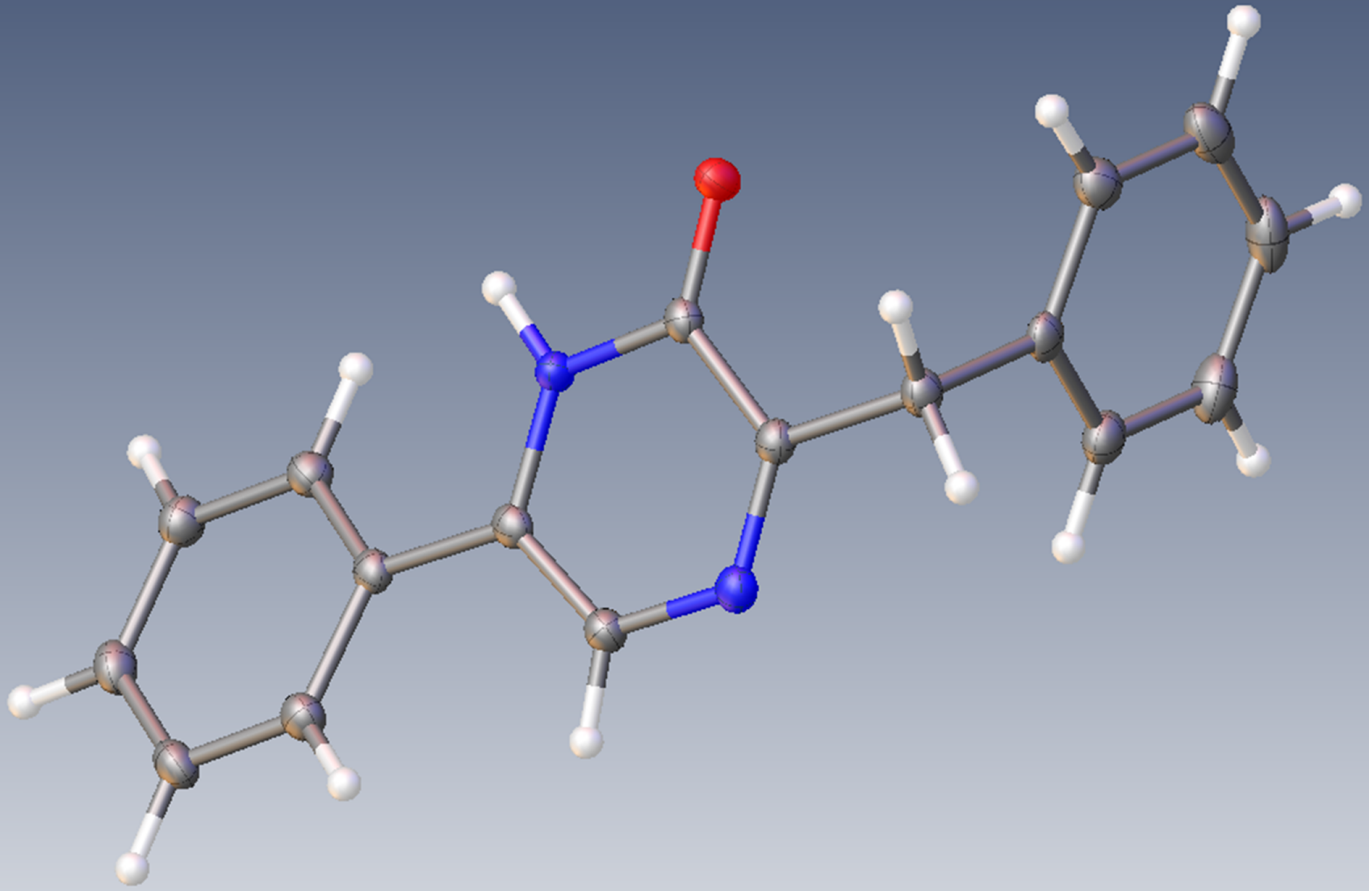
ORTEP depiction of compound **4**{1,2}.

Concerning the structural attribution of the hydroxypyrazines isolated in Table 1 and Table 2, the use of our unambiguous synthetic route to prepare compounds **3**{1,1} [34] or **3**{1,2} [29] secured these issues. However, since a real lack of solubility for isomer **4**{1,2} made it impossible to obtain a complete ^13^C NMR spectrum. On the other hand, following a sample recrystallization from acetic acid, an X-ray derived structure was obtained as seen with the ORTEP depiction in Figure 1. In this structure, the C–O bond length of 1.2425 Å is typical of a double bond thus confirming an oxo-bearing tautomeric form adopted in the solid state.

**Scheme 3:**
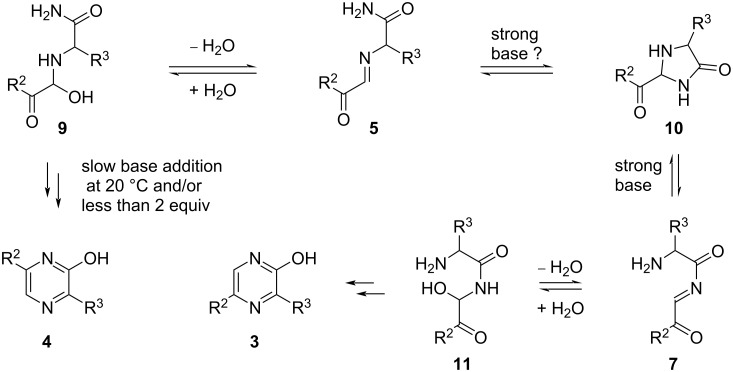
Suggested mechanism for the Reuben G. Jones synthesis of 2-hydroxypyrazines.

Concerning the mechanism of this double condensation, the interpretation of the experimental results must take into account two very likely side reactions. The first one would be a Cannizzaro reaction which, from the glyoxals, would lead to the corresponding α-hydroxyacids. Such transformation has been studied in the past, especially from phenylglyoxal (**1**{1}) [35–36] and since the resulting mandelic acid is water-soluble it would explain why such compounds were not isolated in our experiments. The second side reaction would be the hydrolysis of the α-aminoamides and the resulting α-amino acids would also be water-soluble. For these reasons, a low temperature as well as a plausibly softer base (i.e., tetraalkylammonium hydroxides) would be key parameters to alleviate the impact of these side reactions. These would also explain why a yield increase is observed whenever the glyoxals or the α-aminoamides are used in excess. In each case the reagent in excess would react more quickly to “capture” proportionally more of its partner and diminish the impact of one of these side reactions. On the actual mechanism which leads to the counterintuitive 3,5-substituted-2-hydroxypyrazine isomer **3**, we suggest the occurrence of the imidazolinone **10** (Scheme 3). This reaction intermediate would stem from imine **5** and then, but only under strongly basic conditions, a ring-opening process would lead to the conjugated iminoimide **7**. From this intermediate, the ensuing cyclization, via a hydrated form **11**, would then lead to the isomer **3**. In the absence of a strong base, intermediate **5**, the most likely condensation product between a glyoxal and an α-aminoamide, could only decompose or cyclize (via intermediate **9**) to give isomer **4**. Moreover, when using an excess of a strong base, a low temperature would favor the occurrence of the iminoimide **7** whereas at a higher temperature, decomposition and the cyclization process giving isomer **4** would prevail.

## Conclusion

**Scheme 4:**
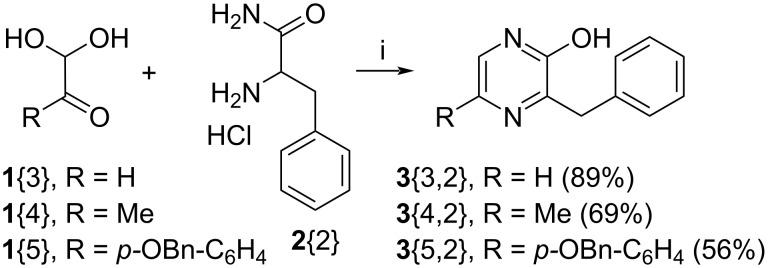
Tetraethylammonium hydroxide-mediated condensation of glyoxal (**1**{3}), methylglyoxal (**1**{4}), or 2-(4-(benzyloxy)phenyl)-2-oxoacetaldehyde (**1**{5}) with phenylalanine amide hydrochloride (**2**{2}). Conditions: i: a) Et_4_NOH, H_2_O/MeOH, −78 to 20 °C; b) 37% HCl.

This report describes some of the reaction parameters which orient and improve the double condensation between phenylglyoxal and two α-aminoamides upon treatment with a base. Aside from confirming previously reported observations concerning the initial temperature and base addition rate [6,13,32], the most prominent result of this study is the discovery that the use of tetraalkylammonium hydroxides as bases does improve the reaction yield, especially when starting from phenylalanine amide (**2**{2}). Moreover, the recourse to chromatography over silica gel at 60 °C was a crucial experimental setting to properly separate and characterize the two isomers occurring. Mechanism-wise the suggestion depicted in Scheme 3 is an attempt to account for all the experimental facts accumulated in the course of this work. In conclusion, we hope that this study will provide elements to extend the use of this reaction to the synthesis of original 2-hydroxypyrazines and/or to the production of large amounts of such synthetic intermediates. As an illustration depicted in Scheme 4, the tetraethylammonium hydroxide-triggered condensation of either glyoxal (**1**{3}), methylglyoxal (**1**{4}), or the readily accessible [37] 2-(4-(benzyloxy)phenyl)-2-oxoacetaldehyde (**1**{5}) with phenylalanine amide hydrochloride (**2**{2}) gave unprecedented yields of the hydroxypyrazines **3**{3,2}, **3**{4,2}, or **3**{5,2} along with only traces of the corresponding isomers in the latter two cases.

## Experimental

**X-ray-based structure determination.** As further detailed in Supporting Information File 1, the structural elucidation of compound **4**{1,2} was achieved using the beamline PROXIMA-2 at Synchrotron SOLEIL and the data were deposited at the Cambridge Crystallographic Data Centre (CCDC), deposition number 2155463.

**Chemistry**. ^1^H NMR and ^13^C NMR spectra were recorded on a Bruker Avance 400 spectrometer at 400 MHz and 100 MHz, respectively. Shifts (δ) are given in ppm with respect to the TMS signal and cross-coupling constants (*J*) are given in hertz. Column chromatography was performed either on Merck silica gel 60 (0.035–0.070 mm) or neutral alumina containing 1.5% of added water using a solvent pump and an automated collecting system driven by a UV detector set to 254 nm unless required otherwise. Sample deposition was carried out by absorption of the mixture to be purified on a small amount of the solid phase followed by its deposition on the top of the column. For chromatography run at 60 °C a complete and illustrated description of the experimental set up is provided in Supporting Information File 1. The low-resolution mass spectra were obtained on an Agilent 1200 series LC/MSD system using an Agilent Jet-Stream atmospheric electrospray ionization system and the high-resolution mass spectra (HRMS) were obtained using a Thermo Fisher Q Exactive Orbitrap. When specified, the anhydrous solvents used were purchased. Experiments under inert atmosphere were carried out by purging the glassware with a stream of dry argon. Then, an argon balloon, fitted with a needle, was used to insure a positive pressure of inert gas during the reaction. Unless stated otherwise, a purity of at least 95% was obtained for all the compounds by means of chromatography or recrystallization and this level of purity was established by TLC, LC–MS, and NMR spectroscopy.

**General protocol for the experiments described in**Table 1, description of entry 10. In a double-necked flask, phenyl glyoxal hydrate (2 g, 0.013 mol) and alanine amide hydrochloride (1.63 g, 0.013 mmol) were dispersed in methanol (35 mL). This was cooled to −78 °C using an ethanol and dry ice bath, under a calcium chloride-protected atmosphere. Then, 20% tetraethylammonium hydroxide (24,12 g, 0.032 mol) was added dropwise by adding the solution into an open-ended syringe fitted with a 0.8 mm needle planted through a rubber stopper placed above the cooled solution. As mentioned in Table 1, the addition rate was calculated to be 2.2 mmol/min. The stirring was continued at −78 °C for 5 minutes after the end of this addition, then the cooling bath was removed, and the resulting solution was stirred for two hours. The mixture was made acidic with 37% hydrochloric acid (4.4 mL, 0.052 mol), diluted in water, stirred for 15 minutes, cautiously made basic with a saturated solution of sodium hydrogen carbonate, and extracted with ethyl acetate (3 × 50 mL). The organic phase was washed with water (50 mL), brine (30 mL), dried over magnesium sulfate, and concentrated to dryness. A chromatography over silica gel (cyclohexane/ethyl acetate 55:45) at 60 °C gave first compound **4**{1,1} (0.08 g, 3%) and then compound **3**{1,1} (1.86 g, 76%) both as white powders.

**3-Methyl-5-phenylpyrazin-2-ol** (**3**{1,1}): NMR data were identical with those observed for the substance obtained via aromatization of 3-methyl-6-phenylpiperazin-2-one [34]. ^1^H NMR (DMSO-*d*_6_) δ 12.28 (br s, 1H), 7.85 (m, 3H), 7.40 (m, 2H), 7.28 (m, 1H), 2.37 (s, 3H); ^13^C NMR (DMSO-*d*_6_) δ 156.3, 155.8, 136.5, 131.3, 129.0, 127.6, 124.9, 122.2, 20.9.

**3-Methyl-6-phenylpyrazin-2-ol** (**4**{1,1}): HRMS (*m*/*z*): [M + H]^+^ calcd for C_11_H_11_N_2_O, 187.0866; found, 187.0864; ^1^H NMR (CD_3_COOD) δ 7.85 (s, 1H), 7.80–7.77 (m, 2H), 7.58–7.54 (m, 3H), 2.50 (s, 3H); ^13^C NMR (CD_3_COOD) δ 158.5, 155.1, 137.5, 130.4, 130.3, 129.3, 126.4, 121.2, 18.0. Note: in some cases, the chromatography fraction containing compound **4**{1,1} had to be recrystallized from toluene.

**General protocol for the experiments described in**Table 2, description of entry 20. In a double-necked flask, phenyl glyoxal hydrate (1.5 g, 9.8 mmol) and phenylalanine amide hydrochloride (1.97 g, 9.8 mmol) were dispersed in methanol (26 mL). This was cooled to −78 °C using an ethanol and dry ice bath, under a calcium chloride-protected atmosphere. Then, 20% tetraethylammonium hydroxide (18,1 g, 24.57 mmol) was added dropwise by adding the solution to an open-ended syringe fitted with a 0.8 mm needle planted through a rubber stopper placed above the cooled solution. As mentioned in Table 2, the addition rate was calculated to be 2.0 mmol/min. The stirring was continued at −78 °C for 5 minutes after the end of this addition, then the cooling bath was removed, and the resulting solution was stirred for three hours. The mixture was made acidic with 37% hydrochloric acid (3.3 mL, 39.31 mmol), diluted in water (150 mL), and stirred for 15 minutes. The precipitate was filtered, washed with water, and dried under vacuum at 60 °C. A chromatography over silica gel of this solid (cyclohexane/ethyl acetate 2:1) at 60 °C gave first a fraction containing compound **4**{1,2} which was further purified by dispersion in a boiling mixture of toluene and cyclohexane (0.09 g, 3.5%) and then compound **3**{1,2} (1.71 g, 66%) both as white powders.

**3-Benzyl-5-phenylpyrazin-2-ol** (**3**{1,2}): NMR data were identical with those reported [29].

**3-Benzyl-6-phenylpyrazin-2-ol** (**4**{1,2}): HRMS (*m*/*z*): [M + H]^+^ calcd for C_17_H_15_N_2_O, 263.1178; found, 263.1179. ^1^H NMR (DMSO-*d*_6_) δ 12.27 (br s, 1H), 7.82 (m, 3H), 7.48 (m, 3H), 7.30 (m, 4H), 7.20 (m, 1H), 4.05 (s, 2H). ^13^C NMR (DMSO-*d*_6_): not sufficiently soluble to detect all the signals. An X-ray-based structure confirmation is described in Supporting Information File 1 and the ORTEP depiction is shown in Figure 1.

**Preparation of 3-benzylpyrazin-2-ol** (**3**{3,2}). A 40% solution of glyoxal (1.56 g, 10.8 mmol) and phenylalanine amide hydrochloride (1.97 g, 9.8 mmol) were dispersed in methanol (26 mL). This was cooled to −78 °C using an ethanol and dry ice bath, under a calcium chloride-protected atmosphere. Then, 20% tetraethylammonium hydroxide (18,1 g, 24.57 mmol) was added dropwise by adding the solution to an open-ended syringe fitted with a 0.8 mm needle planted through a rubber stopper placed above the cooled solution in 16 minutes. The mixture was stirred 5 minutes at −78 °C, then the cooling bath was removed, and the resulting solution was stirred for two hours. This was made acidic with 37% hydrochloric acid (3.3 mL, 39.31 mmol), diluted in water (150 mL), and stirred for 15 minutes. A saturated solution of sodium hydrogencarbonate was then slowly added to bring the pH to 6, this was extracted with ethyl acetate (3 × 50 mL) and the organic layer was washed with brine (30 mL), dried over magnesium sulfate, and concentrated to dryness to give pure compound **3**{3,2} (1.64 g, 89%) as a white powder. HRMS (*m*/*z*): [M + H]^+^ calcd for C_11_H_11_N_2_O, 187.0866; found, 187.0867; ^1^H NMR (DMSO-*d*_6_) δ 12.15 (br s, 1H), 7.29–7.25 (m, 5H), 7.22–7.17 (m, 2H), 3.96 (s, 2H); ^13^C NMR (DMSO-*d*_6_) δ 158.9, 156.0, 139.5, 129.5, 128.6, 126.6, 126.3, 122.6, 39.1.

**Preparation of 3-benzyl-5-methylpyrazin-2-ol** (**3**{4,2}). A 40% solution of methylglyoxal (1.94 g, 10.8 mmol) and phenylalanine amide hydrochloride (1.97 g, 9.8 mmol) were dispersed in methanol (26 mL). This was cooled to −78 °C using an ethanol and dry ice bath, under a calcium chloride-protected atmosphere. Then, 20% tetraethylammonium hydroxide (18,1 g, 24.57 mmol) was added dropwise by adding the solution to an open-ended syringe fitted with a 0.8 mm needle planted through a rubber stopper placed above the cooled solution in 16 minutes. The mixture was stirred 5 minutes at −78 °C, then the cooling bath was removed, and the resulting solution was stirred for two hours. This was made acidic with 37% hydrochloric acid (3.3 mL, 39.31 mmol), diluted in water (150 mL), and stirred for 15 minutes. A saturated solution of sodium hydrogencarbonate was then slowly added to bring the pH to 6, this was extracted with ethyl acetate (3 × 50 mL) and the organic layer was washed with brine (30 mL), dried over magnesium sulfate, and concentrated to dryness. The residue was purified by a chromatography over silica gel (cyclohexane/ethyl acetate 2:1; a 10-fold increased UV detector sensitivity is recommended) at 60 °C and compound **3**{4,2} was obtained as a white powder (1.37 g, 69%). HRMS (*m*/*z*): [M + H]^+^ calcd for C_12_H_13_N_2_O, 201.1022; found, 201.1023; ^1^H NMR (DMSO-*d*_6_) δ 11.95 (br s, 1H), 7.28–7.24 (m, 4H), 7.19–7.15 (m, 1H), 7.13 (m, 1H), 3.95 (s, 2H), 2.15 (s, 3H); ^13^C NMR (DMSO-*d*_6_) δ 157.0, 155.2, 138.7, 130.9, 129.4, 128.7, 126.6, 123.6, 38.7, 9.5.

**Preparation of 3-benzyl-5-(4-(benzyloxy)phenyl)pyrazin-2-ol** (**3**{5,2}). The crude 2-(4-(benzyloxy)phenyl)-2-oxoacetaldehyde (**1**{5}), prepared as described on page 15 of a patent [37], (2.96 g, 0.011 mol) and phenylalanine amide hydrochloride (2.30 g, 0.011 mol) were dispersed in methanol (30 mL). This was cooled to −78 °C using an ethanol and dry ice bath, under a calcium chloride-protected atmosphere. Then, 20% tetraethylammonium hydroxide (20,12 g, 0.028 mol) was added dropwise by adding the solution to an open-ended syringe fitted with a 0.8 mm needle planted through a rubber stopper placed above the cooled solution in 17 minutes. The mixture was stirred 5 minutes at −78 °C, then the cooling bath was removed, and the resulting solution was stirred for two hours. This was made acidic with 37% hydrochloric acid (3.3 mL, 39.31 mmol), diluted in water (150 mL), and stirred for 15 minutes. The precipitate was filtered, washed with water, and dried under vacuum at 60 °C. The resulting solid was dispersed in boiling ethanol (100 mL) containing 37% hydrochloric acid (3 mL), the suspension was left to cool, filtered, the solid was washed with water, ethanol, and dried under vacuum at 60 °C to give pure compound **3**{5,2} (2.39 g, 56%) with analytical data identical with those previously reported [29].

## Supporting Information

File 1 Images of the experimental set up to run chromatography at 60 °C, a description of the crystallization, data collection, for the structural determination of compound **4**{1,2} as well as copies of the ^1^H and ^13^C NMR spectra of all compounds described.
