# Supplementary material for: On Reuben G. Jones synthesis of 2-hydroxypyrazines
Source: Beilstein J Org Chem. 2022 Jul 29;18:935–43. doi: 10.3762/bjoc.18.93 (PMC9344544; doi:10.3762/bjoc.18.93)

BEILSTEIN JOURNAL OF ORGANIC CHEMISTRY

Supporting Information

for

On Reuben G. Jones synthesis of 2-hydroxypyrazines

Pierre Legrand and Yves L. Janin

Beilstein J. Org. Chem. **2022,** *18,* 935–943. doi:10.3762/bjoc.18.93

Images of the experimental set up to run chromatography at 60 °C, a description of the crystallization, data collection, for the structural determination of compound 4{1,2} as well as copies of the ^{1}H and ^{13}C NMR spectra of all compounds described

License and Terms: This is a supporting information file under the terms of the Creative Commons Attribution License (https://creativecommons.org/licenses/by/4.0). Please note that the reuse, redistribution and reproduction in particular requires that the author(s) and source are credited and that individual graphics may be subject to special legal provisions.
The license is subject to the *Beilstein Journal of Organic Chemistry* terms and conditions: (https://www.beilstein-journals.org/bjoc/terms)

Experimental set up to run chromatography at 60 °C

As depicted, double enveloped columns were used to maintain a 60 °C temperature with a heated water circulation on the outside. Moreover, the eluent bottles from which the solvent was pumped into the column, were placed in the heated water bath of the apparatus (a Bioblock polystat 12HT) insuring this water circulation. Note: Pumping the eluent up to one meter at 60 °C without encountering too much cavitation problems turns out to be possible with mixtures of ethyl acetate and cyclohexane.

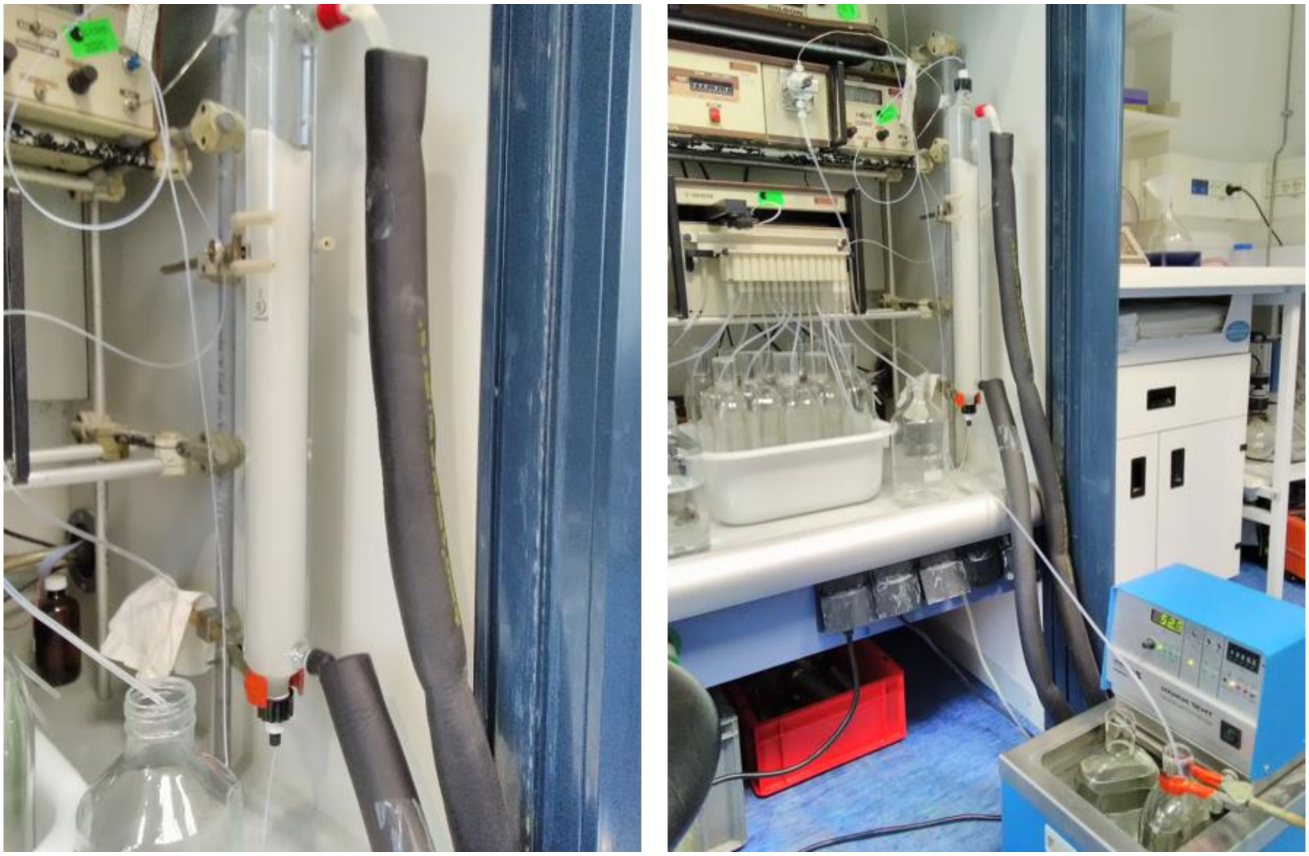

Experimental setup to undertake a chromatography at 60 °C (at 52.5 °C on the right picture). Left: picture of a double enveloped column charged with silica gel and a hot water circulation on the outside. Right: picture of the entire setup, starting with the water heating apparatus set on the floor which pumps the hot water in the column via the two insulated tubes, the two solvent bottles warmed by the hot water bath and the PTFE tube placed in one of these bottles allows to pump it to the top of the column.

Crystallization, data collection, and structure determination

Suitable crystals of compound **4**{1,2} were obtained by recrystallization from acetic acid. Crystals were then flash-cooled in liquid nitrogen using Paratone-paraffin 50% (v/v) oil as the cryoprotectant. X-ray diffraction data were collected at a wavelength of 0.7293 Å on the beamline PROXIMA-2 at Synchrotron SOLEIL (St. Aubin, France). Diffraction data were integrated and reduced using the XDS program package.[1] Structures were solved using SHELXT[2] and refined with SHELXL.[3]

Parameters for compound **4**{1,2}: Datablock: YJ31780-038-4. Space group: *P*21/*c*. Unit cell parameters: a = 12.424 Å, b = 11.184 Å, c = 9.634 Å, α = 90.00(3)°, β = 107.73(3)°, γ = 90.00(3). CCDC Deposition Number: 2155463. CSD Refcode Entry: NAXMOS. DOI: 10.5517/ccdc.csd.cc2bby2q

References

1. Kabsch, W. *Acta Cryst*. **2010**, *D66*, 125.
2. Sheldrick, G.M. *Acta Cryst.* **2015**, *A71*, 3.
3. Sheldrick, G.M. *Acta Cryst.* **2015**, *C71*, 3.

^{1}H and ^{13}C NMR spectra

3-Methyl-5-phenylpyrazin-2-ol (**3**{1,1})

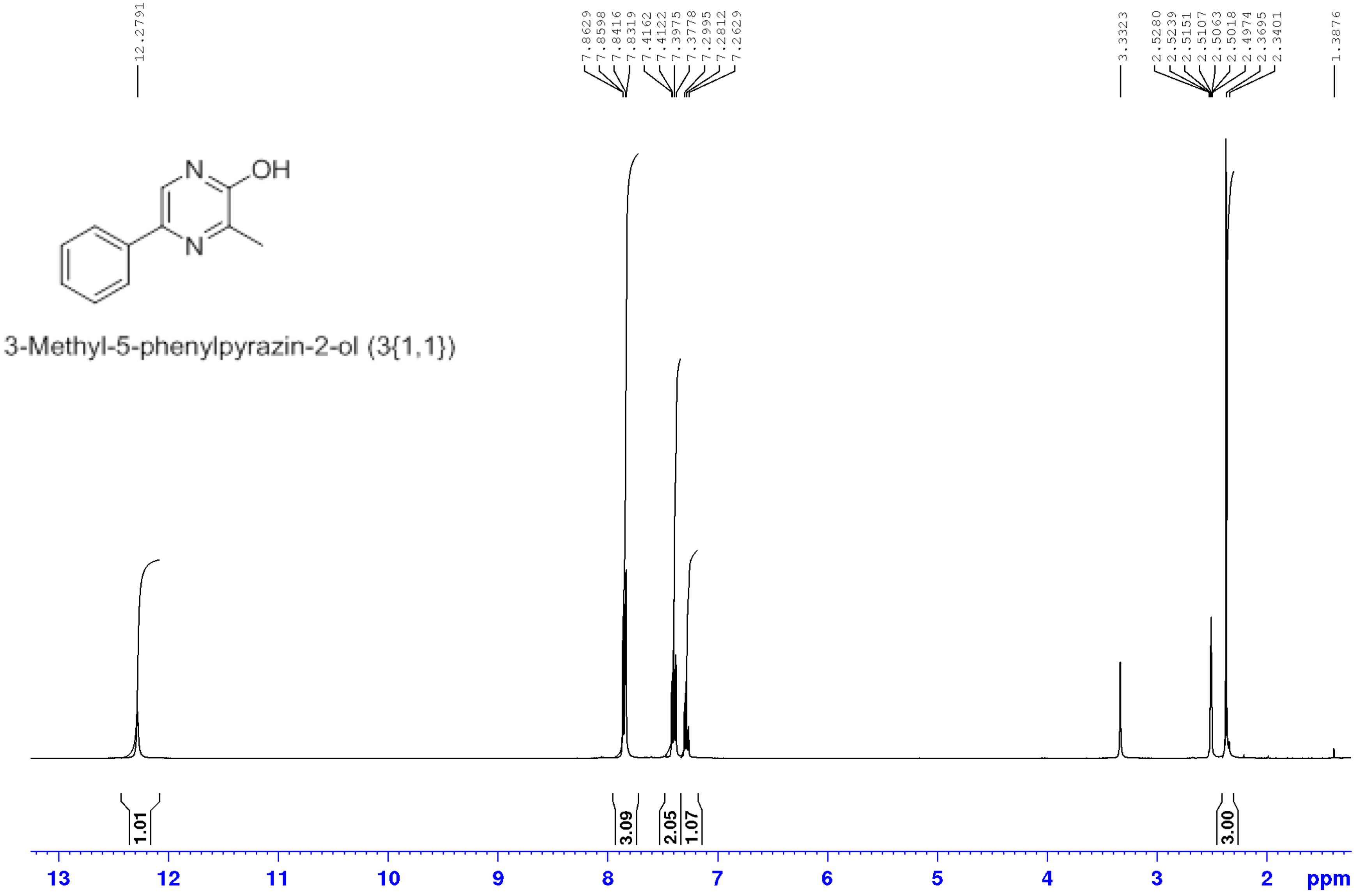

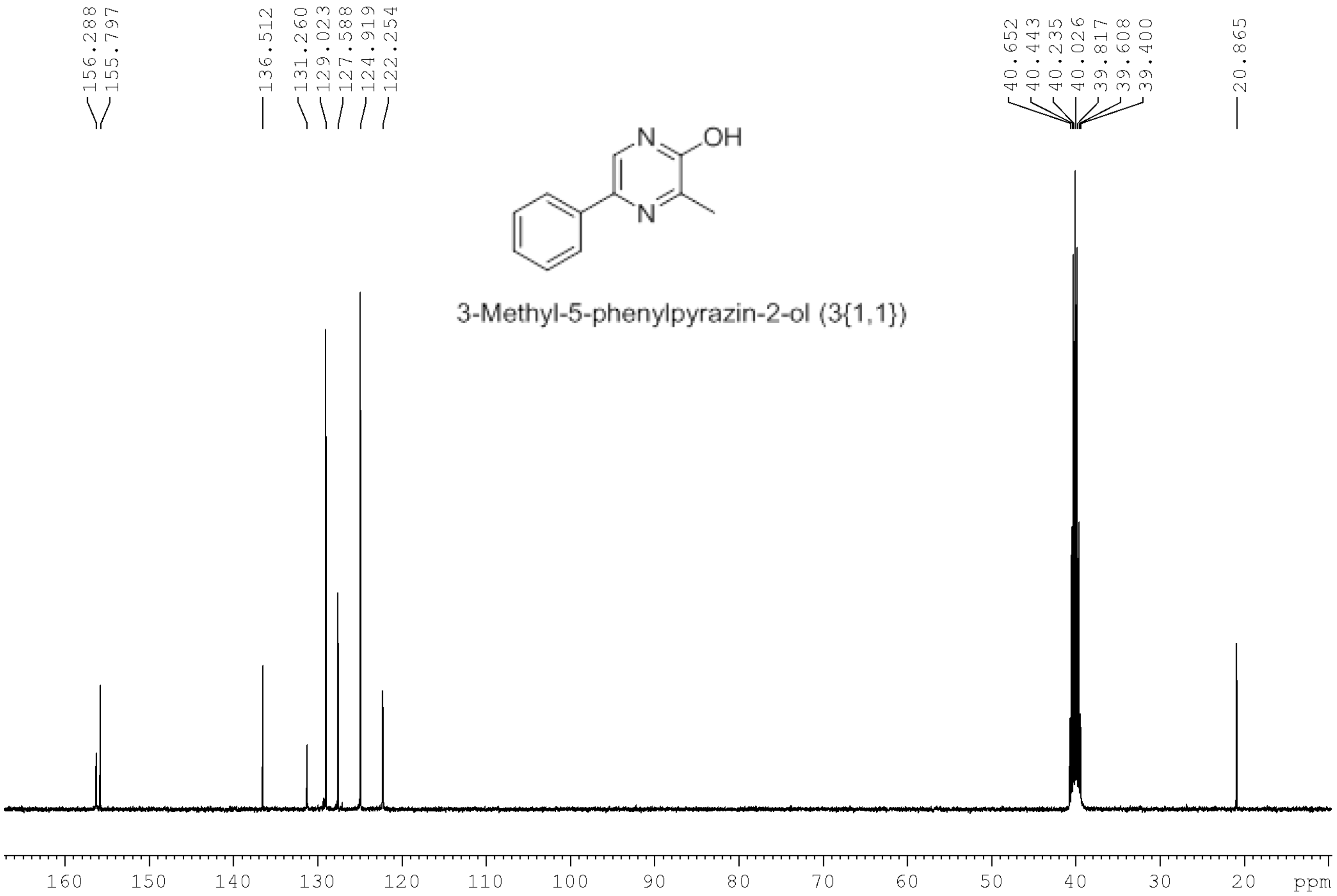

3-Methyl-5-phenylpyrazin-2-ol (3{1,1})

3-Methyl-6-phenylpyrazin-2-ol (**4**{1,1})

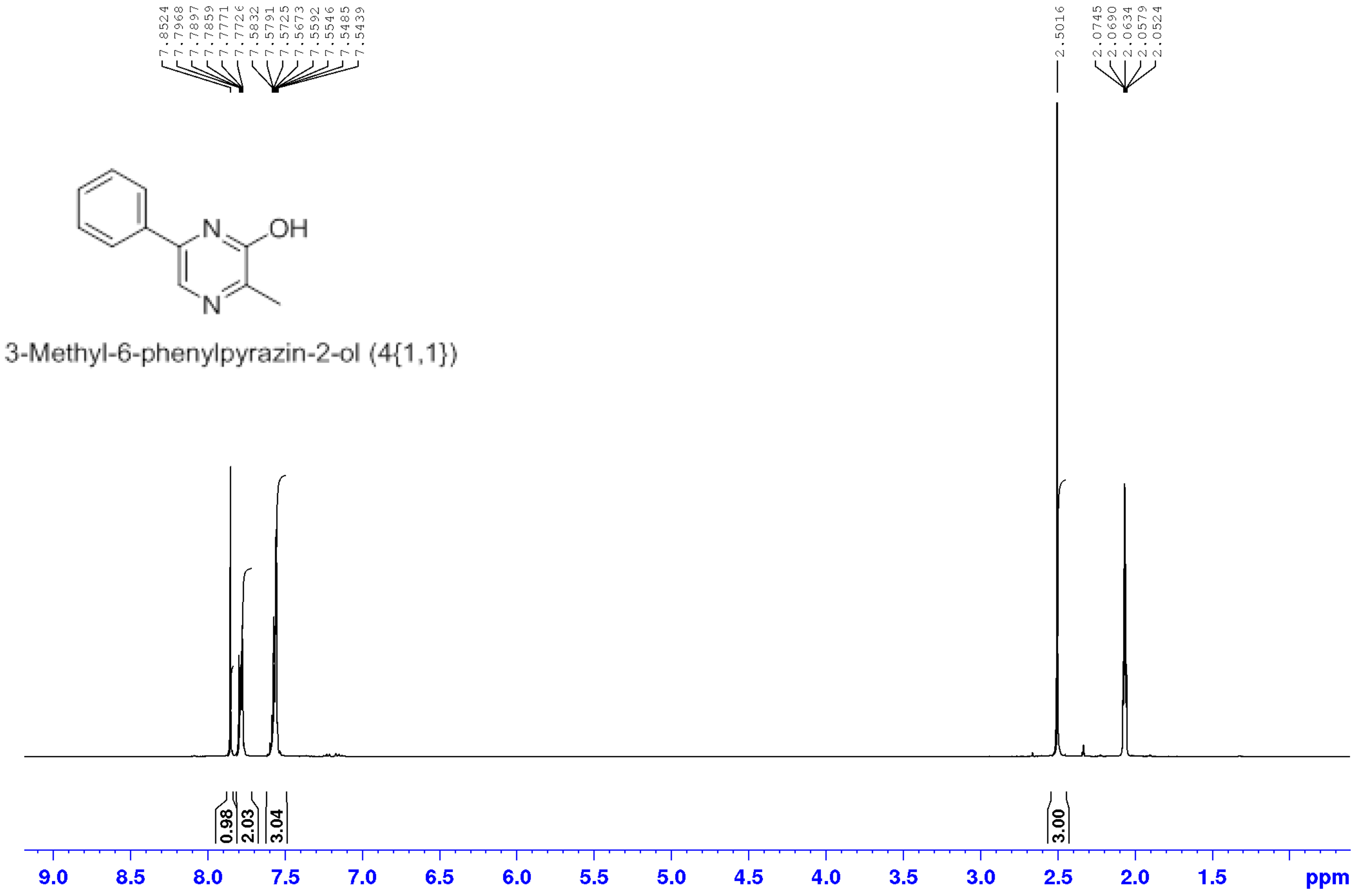

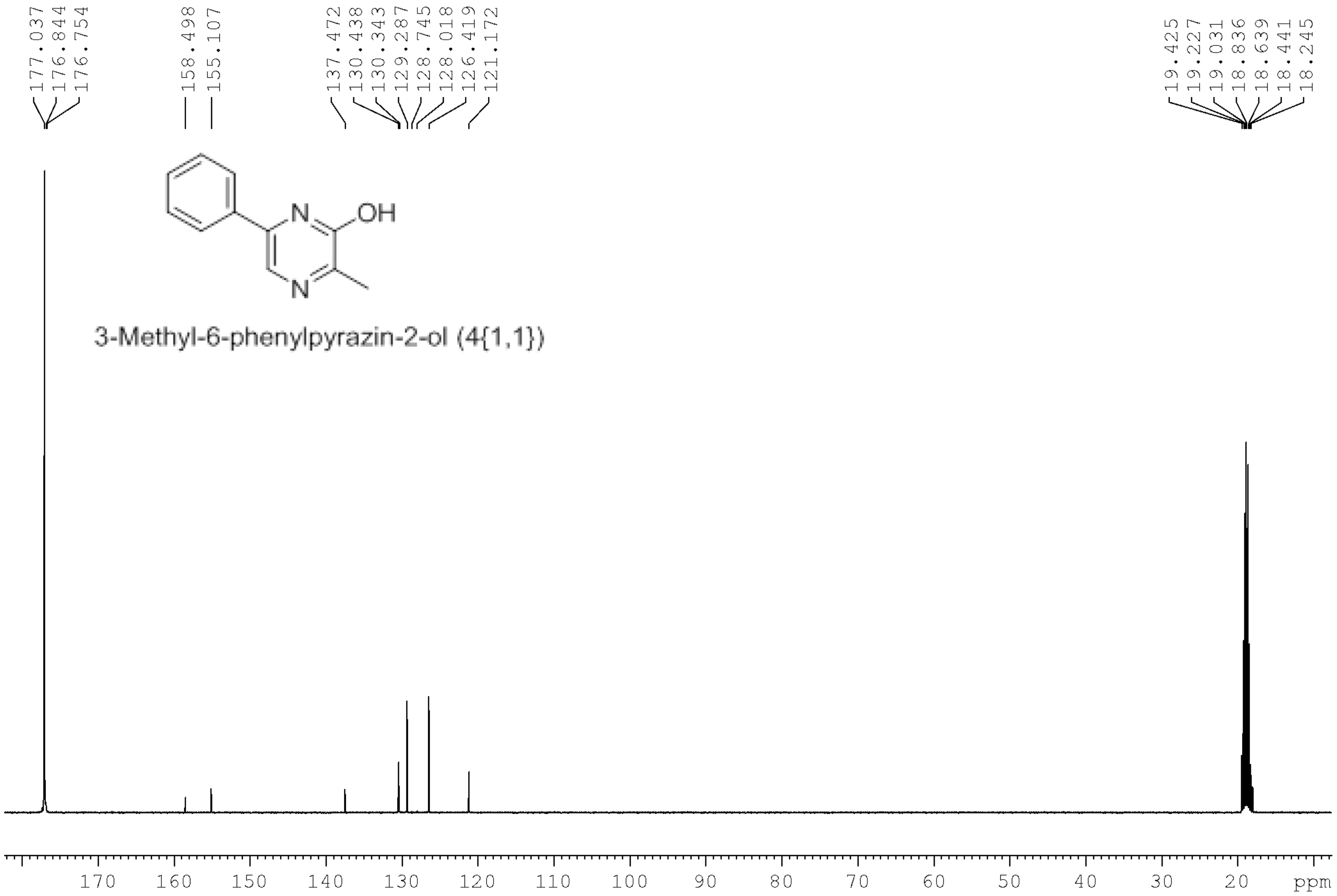

3-Methyl-6-phenylpyrazin-2-ol (4{1,1})

3-Benzyl-5-phenylpyrazin-2-ol (**3**{1,2})

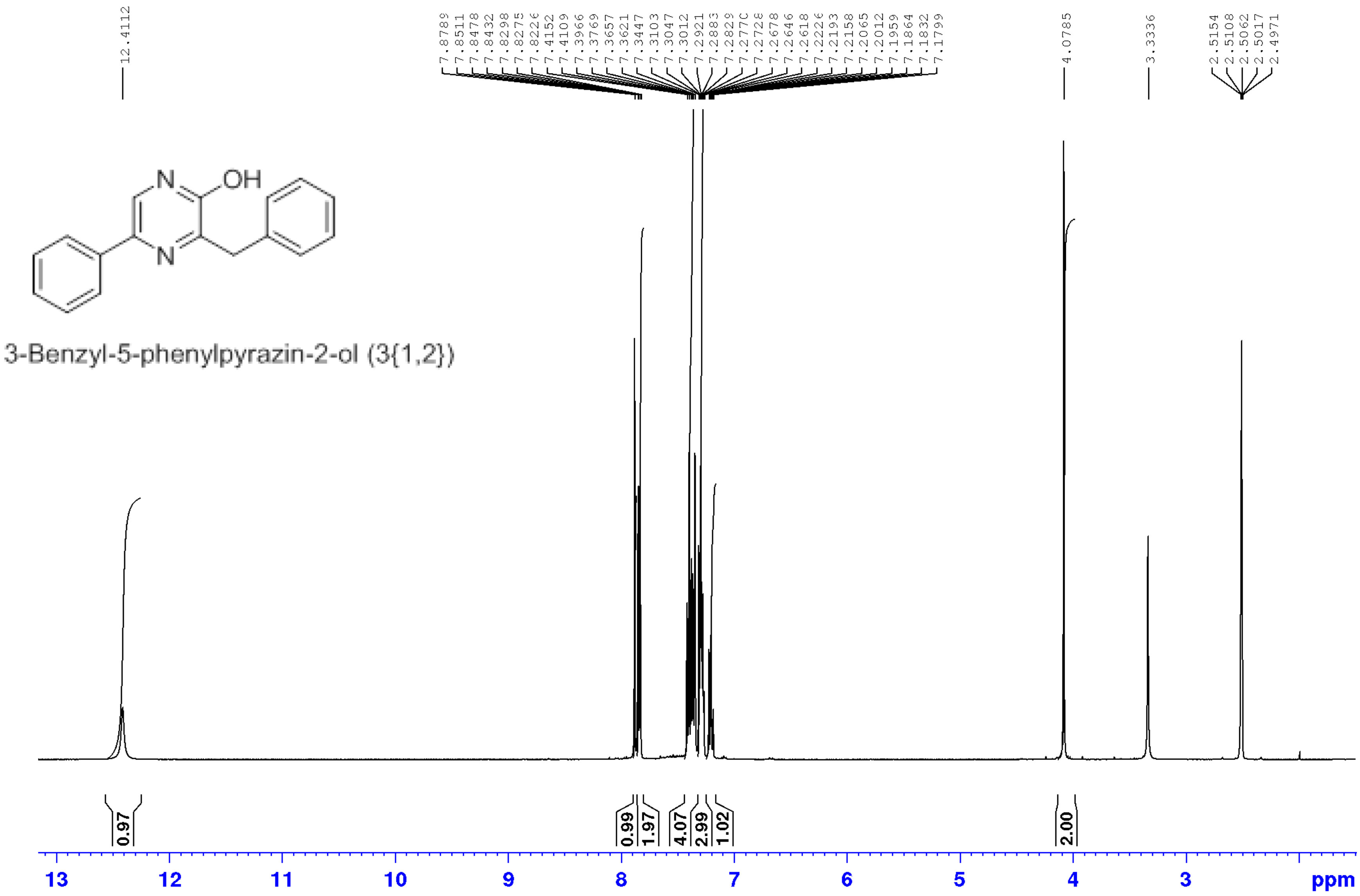

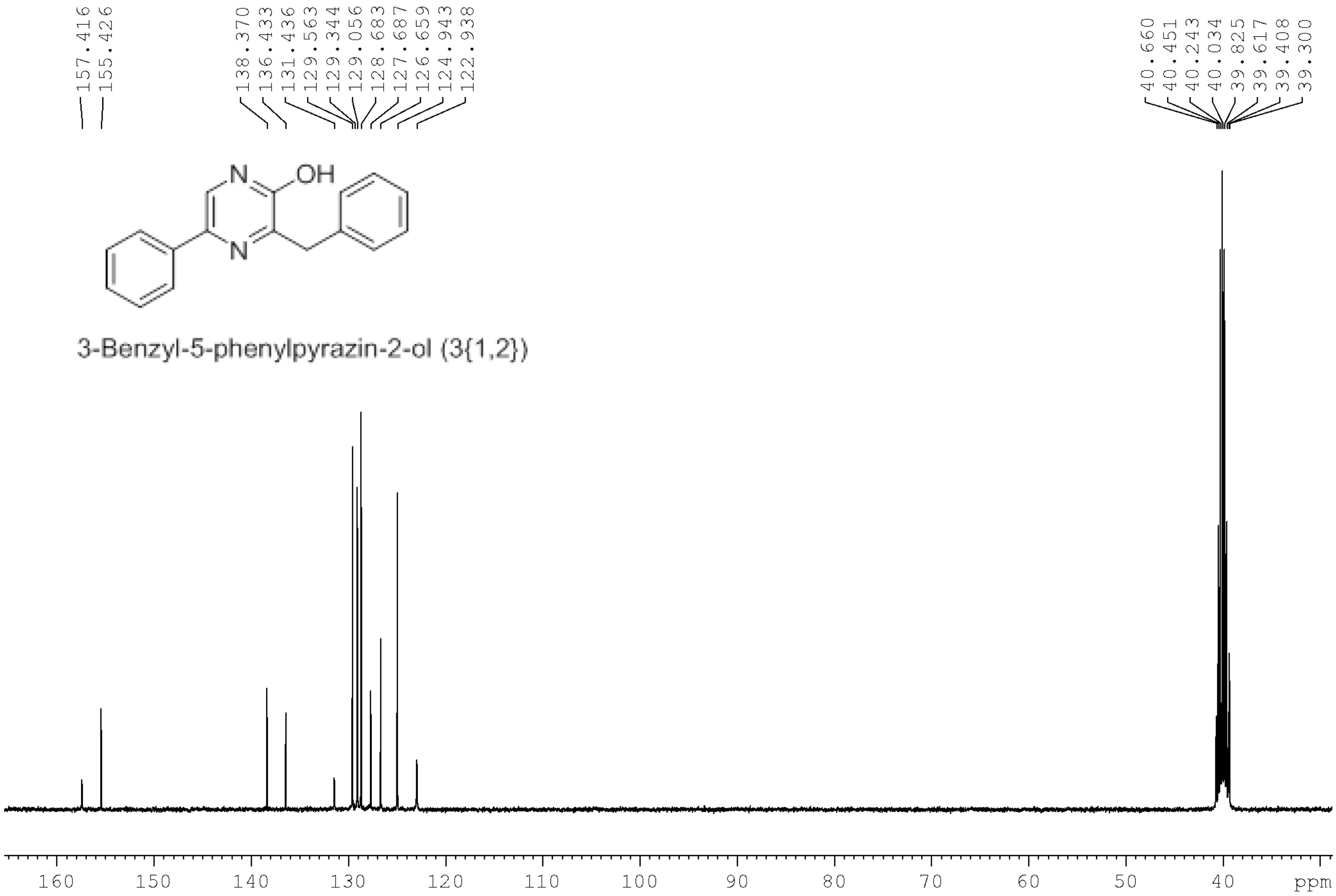

3-Benzyl-5-phenylpyrazin-2-ol (3{1,2})

3-Benzyl-6-phenylpyrazin-2-ol (**4**{1,2})

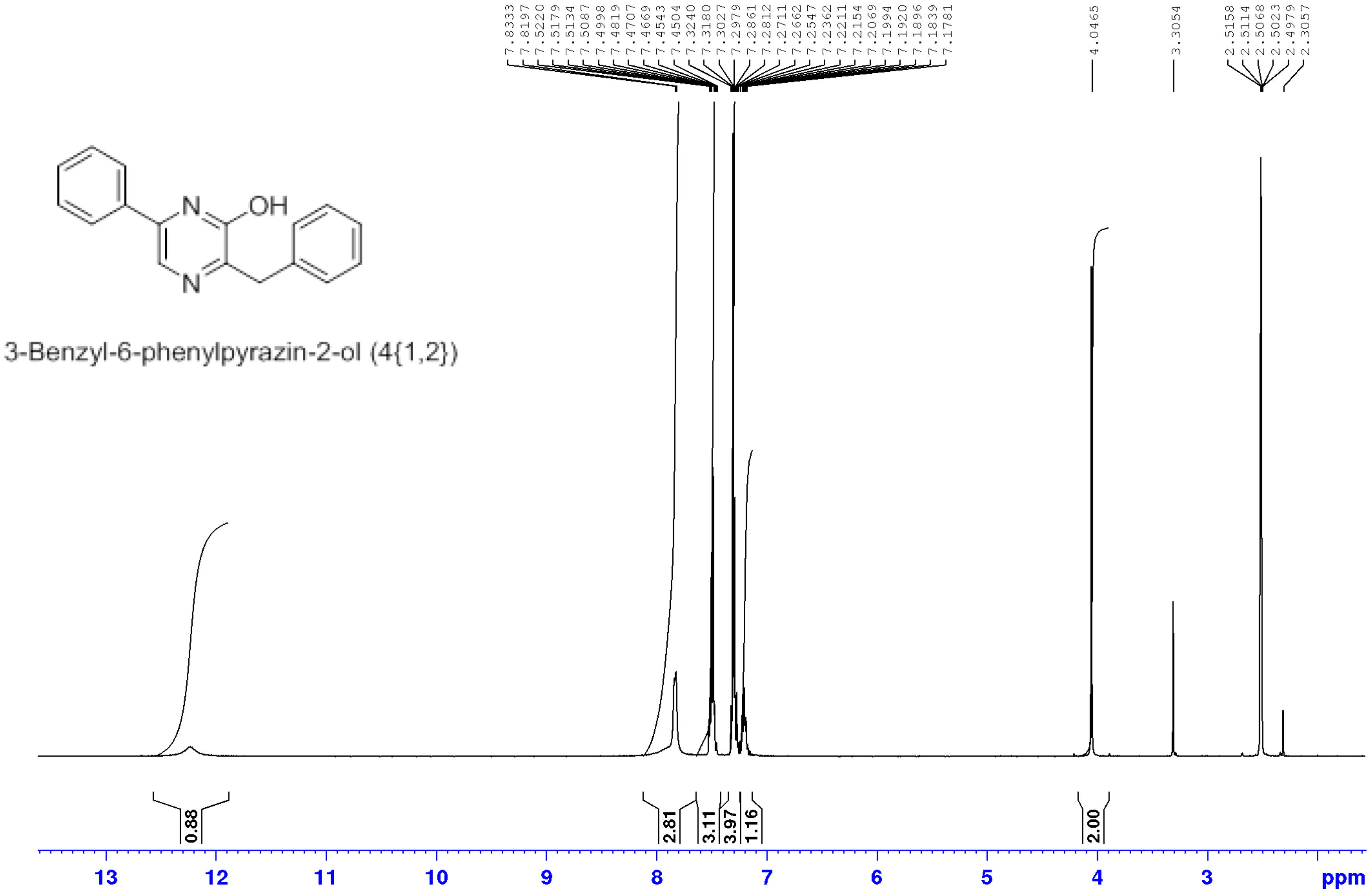

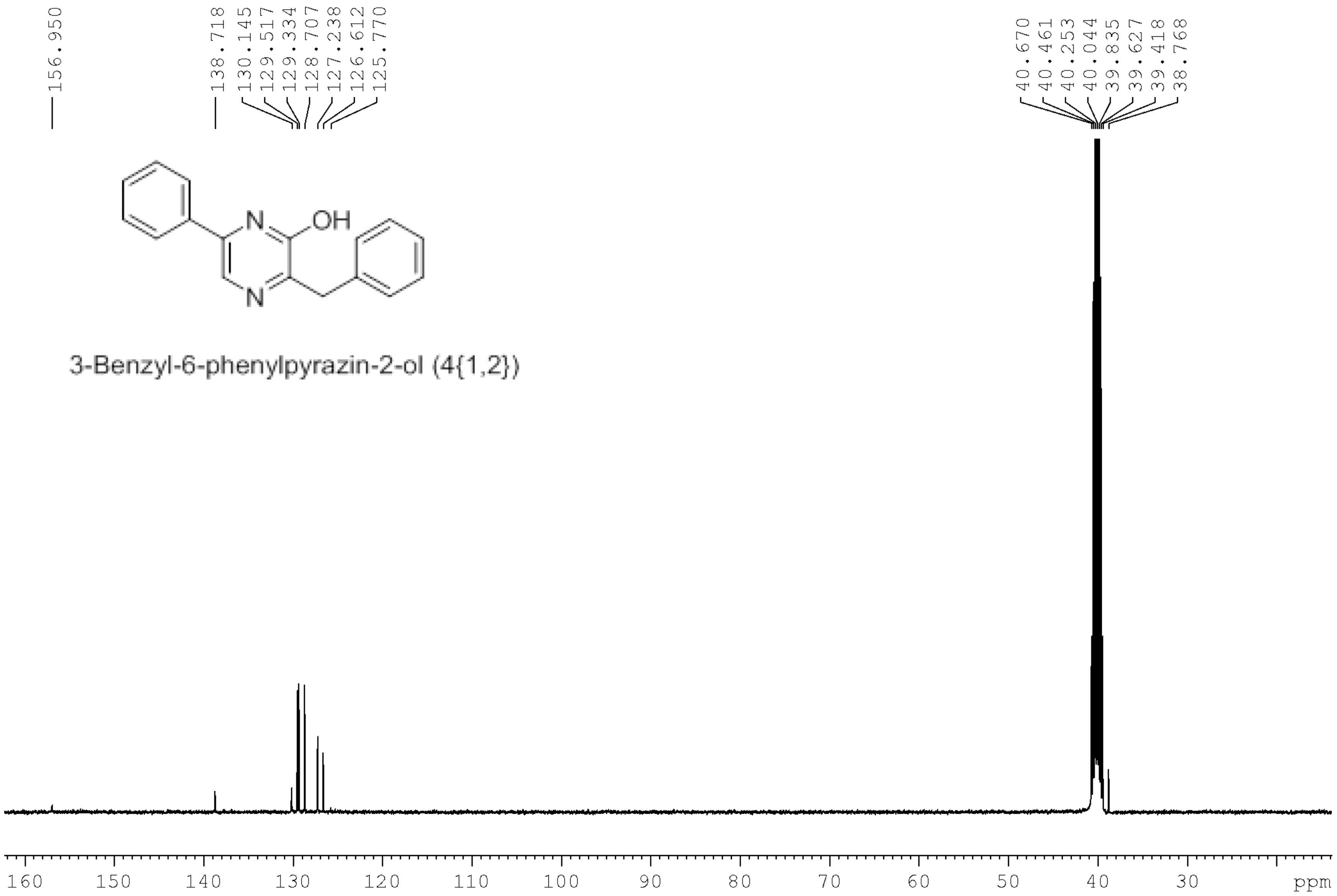

3-Benzyl-6-phenylpyrazin-2-ol (4{1,2})

3-Benzylpyrazin-2-ol (**3**{3,2})

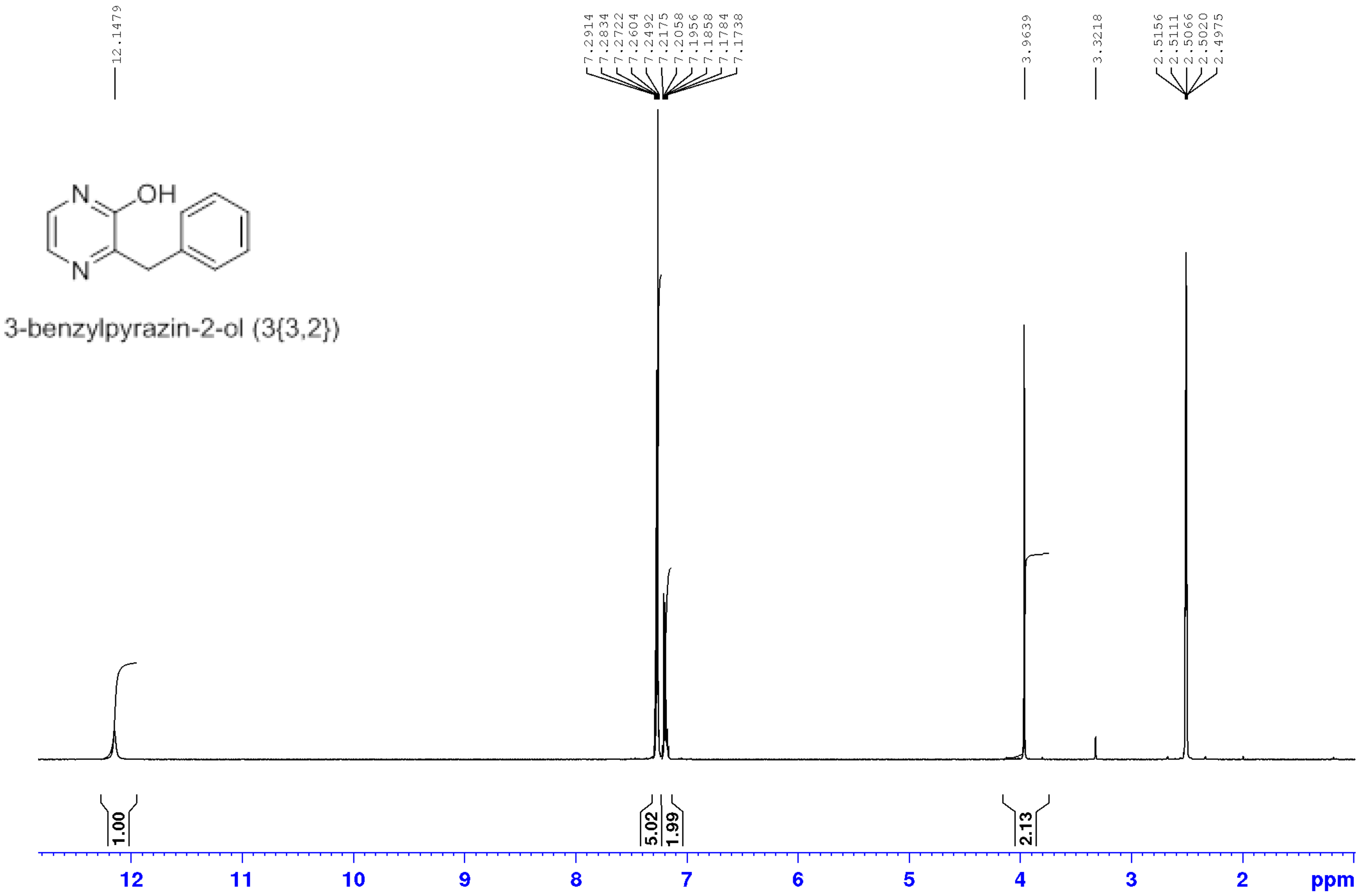

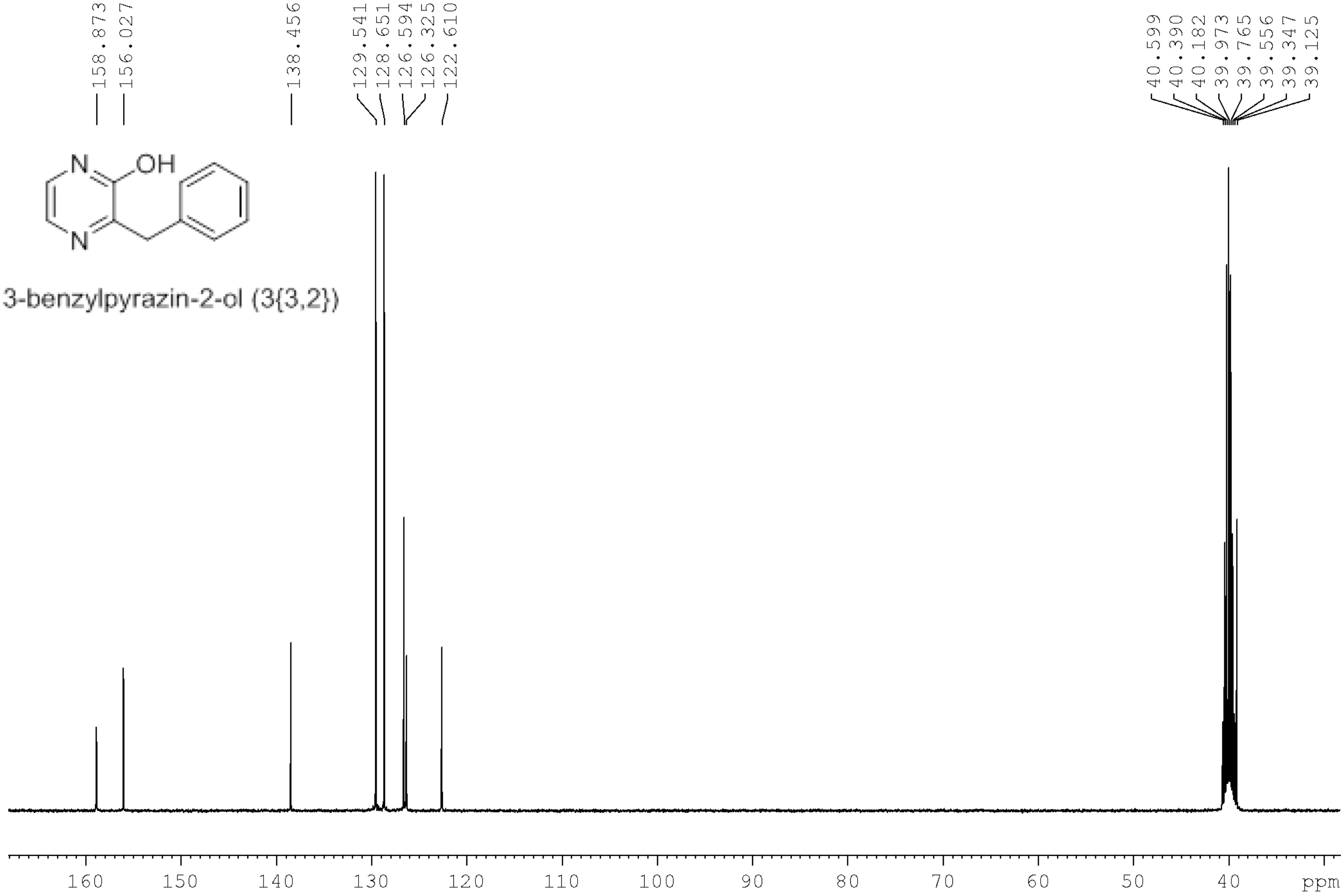
158.873
156.027
138.456
129.541
128.651
126.594
126.325
122.610
40.599
40.390
40.182
39.973
39.765
39.556
39.347
39.125
N
OH
N
3-benzylpyrazin-2-ol (3{3,2})
160 150 140 130 120 110 100 90 80 70 60 50 40 ppm

3-Benzyl-5-methylpyrazin-2-ol (**3**{4,2})

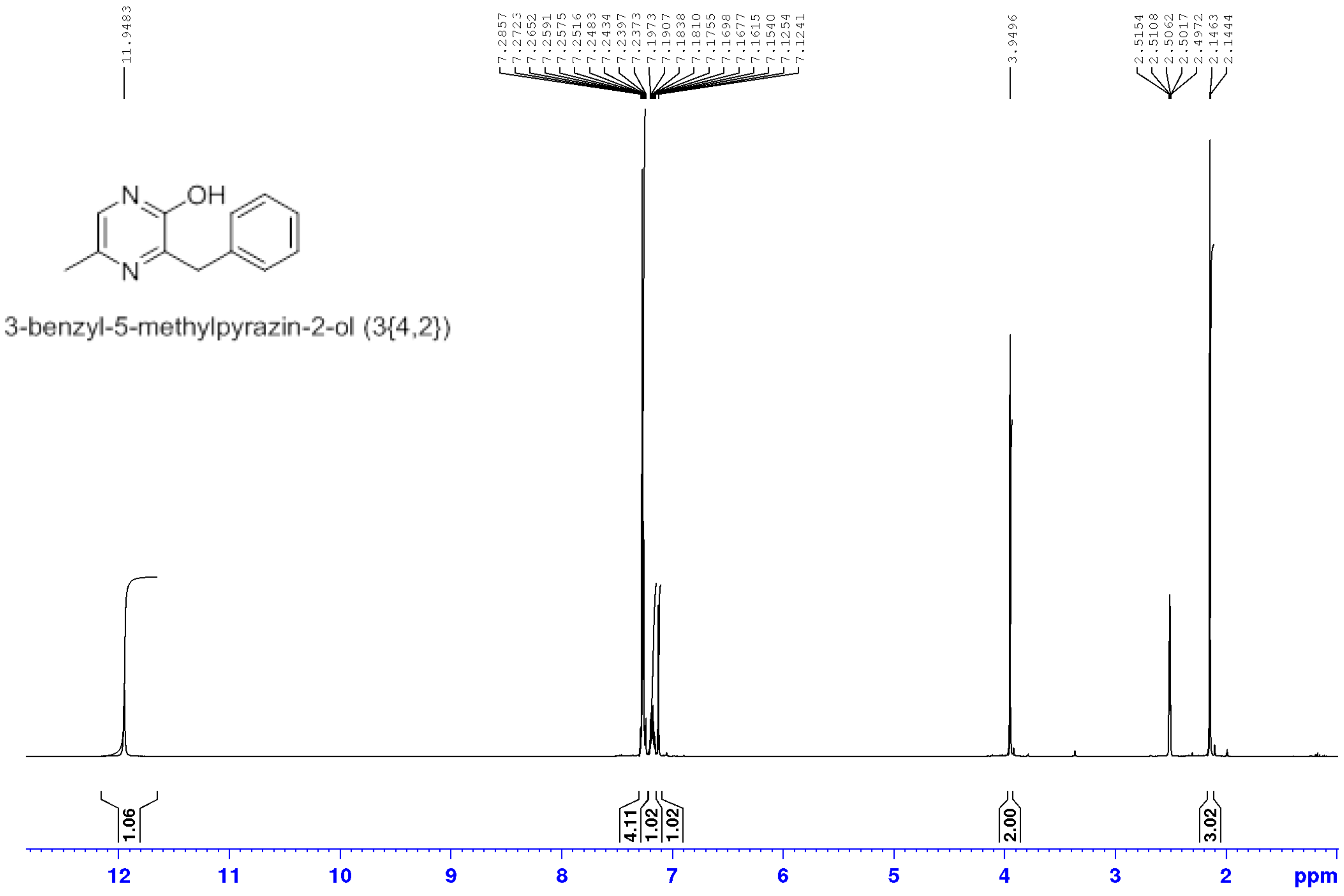

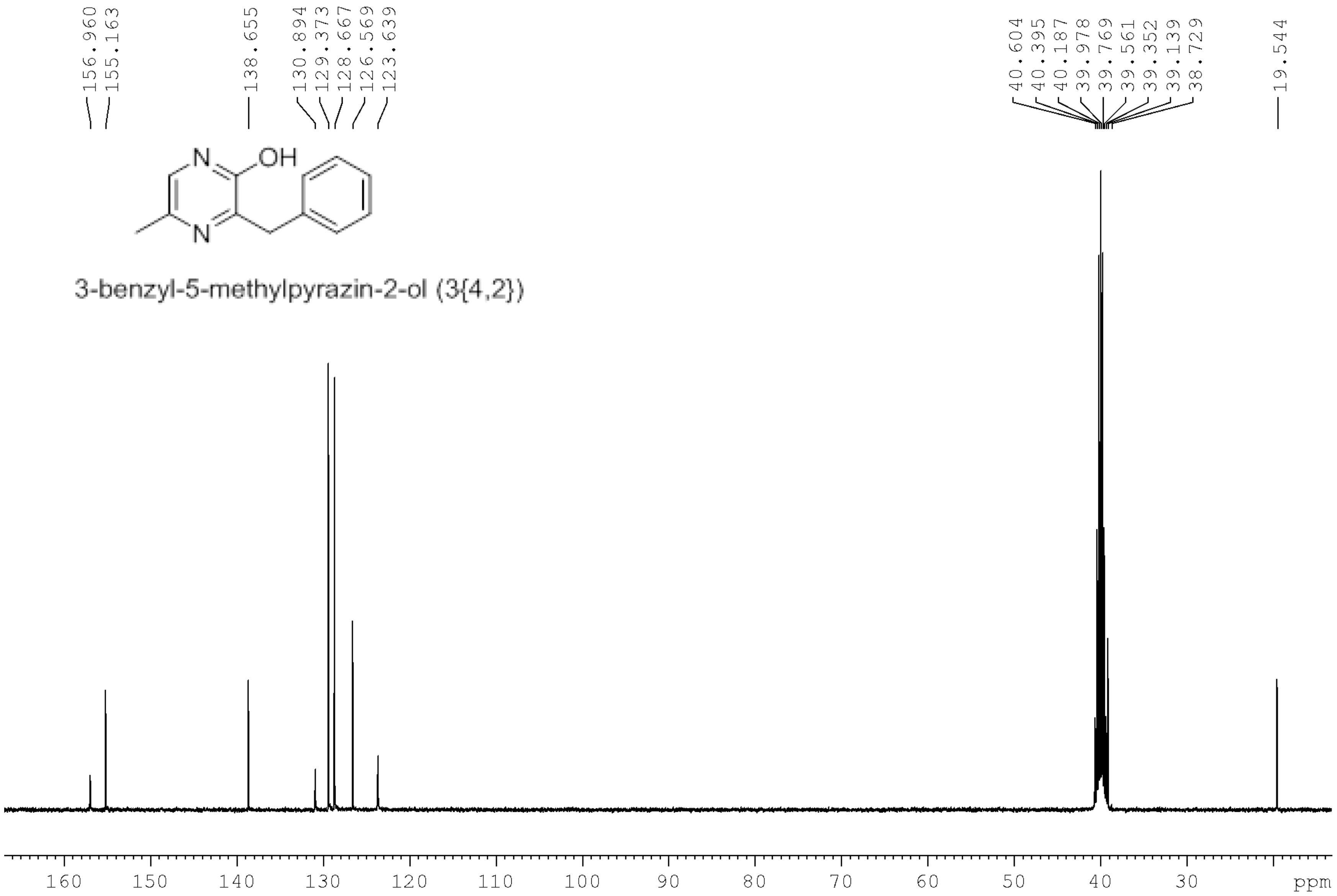

3-benzyl-5-methylpyrazin-2-ol (3{4,2})

3-Benzyl-5-(4-(benzyloxy)phenyl)pyrazin-2-ol (**3**{5,2})

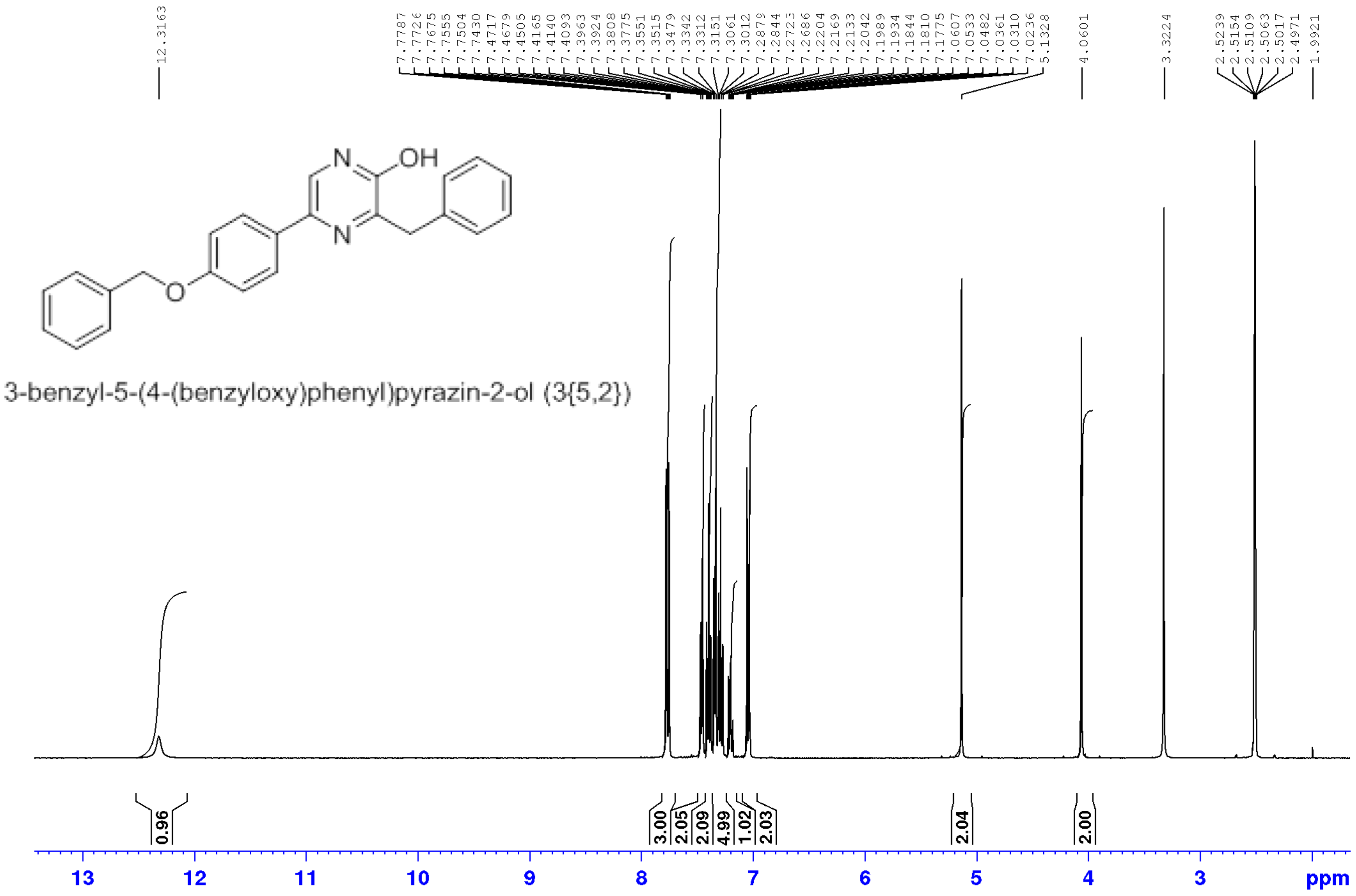

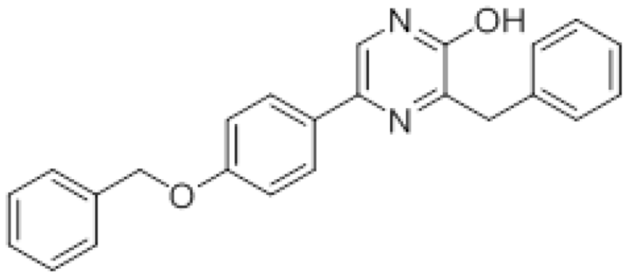

3-benzyl-5-(4-(benzyloxy)phenyl)pyrazin-2-ol (3{5,2})

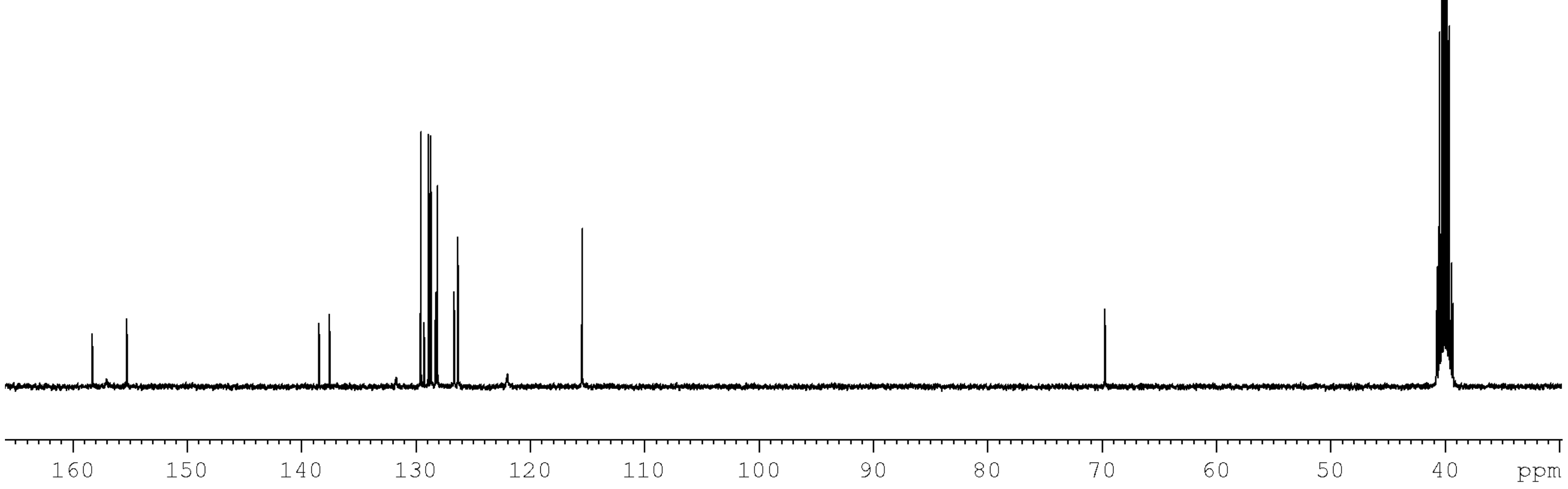

Supplement: File 1 — Images of the experimental set up to run chromatography at 60 °C, a description of the crystallization, data collection, for the structural determination of compound 4{1,2} as well as copies of the 1H and 13C NMR spectra of all compounds described. [file Beilstein_J_Org_Chem-18-935-s001.pdf]
